# Two Legionnaires' disease cases associated with industrial waste water treatment plants: a case report

**DOI:** 10.1186/1471-2334-10-343

**Published:** 2010-12-02

**Authors:** Jaana Kusnetsov, Liisa-Kaarina Neuvonen, Timo Korpio, Søren A Uldum, Silja Mentula, Tuula Putus, Nhu Nguyen Tran Minh, Kari-Pekka Martimo

**Affiliations:** 1 National Institute for Health and Welfare (THL), Water and Health Unit, P.O.Box 95, FI-70701 Kuopio, Finland; 2 Medivire Occupational Health Care Center, Finland; 3 UPM-Kymmene Oyj, Finland; 4 Statens Serum Institut, Copenhagen, Denmark; 5 National Institute for Health and Welfare, Bacteriology Unit, Helsinki, Finland; 6 University of Turku, Turku, Finland; 7 National Institute for Health and Welfare, Epidemiologic Surveillance and Response Unit, Helsinki, Finland; 8 Mehiläinen Occupational Health Care, Helsinki, Finland

## Abstract

**Background:**

Finnish and Swedish waste water systems used by the forest industry were found to be exceptionally heavily contaminated with legionellae in 2005.

**Case presentation:**

We report two cases of severe pneumonia in employees working at two separate mills in Finland in 2006. *Legionella *serological and urinary antigen tests were used to diagnose Legionnaires' disease in the symptomatic employees, who had worked at, or close to, waste water treatment plants. Since the findings indicated a *Legionella *infection, the waste water and home water systems were studied in more detail. The antibody response and *Legionella *urinary antigen finding of Case A indicated that the infection had been caused by *Legionella pneumophila *serogroup 1. Case A had been exposed to legionellae while installing a pump into a post-clarification basin at the waste water treatment plant of mill A. Both the water and sludge in the basin contained high concentrations of *Legionella pneumophila *serogroup 1, in addition to serogroups 3 and 13. Case B was working 200 meters downwind from a waste water treatment plant, which had an active sludge basin and cooling towers. The antibody response indicated that his disease was due to *Legionella pneumophila *serogroup 2. The cooling tower was the only site at the waste water treatment plant yielding that serogroup, though water in the active sludge basin yielded abundant growth of *Legionella pneumophila *serogroup 5 and *Legionella rubrilucens*. Both workers recovered from the disease.

**Conclusion:**

These are the first reported cases of Legionnaires' disease in Finland associated with industrial waste water systems.

## Background

From 1995 until 2007, the number of reported Legionnaires' disease (LD) cases in Finland has varied from 10 up to 31 per year, with an annual incidence of 2-6 cases per million [1]. The reporting of LD and *Legionella*-positive laboratory results has been mandatory for both clinicians and clinical laboratories since 1995. The incidence rate in Finland is much lower compared to the mean incidence rate of LD in European countries, which has been 11 cases per million in 2007 and 2008 [2].

Previous Finnish *Legionella *survey studies revealed that 30% of the hot water systems and 47% of the cooling water systems were contaminated with legionellae [3,4]. In addition, the few Finnish case studies where both clinical and environmental *Legionella *strains were obtained for molecular typing have indicated that hot water systems were the source of infection [5,6].

Following Swedish reports of extremely high concentrations of legionellae in biological waste water treatment plants and LD in an employee working near a plant [7], an environmental study was initiated focusing on waste water systems used by the Finnish paper and pulp industries. In the first part of the study, culturable legionellae were detected in 73% (11/15) of industrial active sludge basins containing waste water, with the highest concentration being 1.9 × 10^9 ^cfu/l (Unpublished data, Kusnetsov J, Torvinen E, Lehtola M and Miettinen IT). In addition, the microscopic PNA-FISH method [8] revealed *Legionella pneumophila *(*L. pneumophila*) cells to be present in all waste water basins (up to 1.7 × 10^10 ^cells/l). After these environmental findings, two cases of LD were diagnosed via the occupational health services of the participating paper and pulp mills. These cases are reported here.

## Case presentation

General awareness of potential *Legionella *exposure has increased recently in the paper and pulp industries. As a consequence, these two severe respiratory infections suffered by employees working in proximity of waste water treatment plants of two different paper and pulp mills were studied in more detail for suspected *Legionella *infection.

### Methods

*Legionella *antigens were detected by urinary antigen immunochromatography (Binax-now, Inc. Portland). Serum antibodies against legionellae were first detected by an in-house EIA-method (TYKSLAB, Turku) and later with an in-house IFA-method (HUSLAB, Helsinki). The EIA-method detects antibodies against *L. pneumophila *serogroups 1 to 4 and *L. micdadei *[9] and the IFA-method detects IgG-, IgA-and IgM-antibodies against *L. pneumophila *serogroups 1 to 8 and *L. gormanii, L. longbeachae, L. dumoffii, L. bozemanii *and *L. micdadei *[10]. The definitions given by the European Working Group for *Legionella *Infections were followed to determine whether these cases were confirmed or presumptive LD cases [11].

At the workplaces, the water samples were analysed before and, in more detail, after these cases were diagnosed. For culture of waste waters, samples were diluted 3-fold before processing according to ISO 11731 [12]. Clean water samples were also diluted in the same way and also concentrated by filtration. Portions of diluted, undiluted and concentrated samples were inoculated directly, acid-washed (pH 2.2, 4 min) or heat-treated (50°C, 30 min) before inoculation onto GVPC medium plates (buffered charcoal yeast extract medium containing glycine, vancomycin, polymyxin B and cycloheximide, Oxoid Ltd, Cambridge, UK). Water samples from the hot and cold water systems of the cases' homes were analysed with the standard method [12], without dilution. Media plates were incubated for 10 days at 36 ± 1°C and colonies resembling legionellae were further confirmed by growth tests according to the standard method [12].

Serotyping of *Legionella *strains was first performed with the Oxoid Legionella Latex Test (DR0800 M, Oxoid). The *L. pneumophila *strains were further serogrouped with the Denka Seiken antisera set (Denka Seiken Co. Ltd, Tokyo, Japan) or the Dresden panel of monoclonal antibodies (MAb) [13]. *L. pneumophila *serogroup 1 strains were further subgrouped using the Dresden monoclonal panel. Non*-pneumophila Legionella *strains were identified to species level by growth and biochemical tests and partial 16 S rRNA sequencing by commercial service at FIMM (FIMM, Helsinki, Finland) using primers fD1 Mod and 533r [14] and GenBank database [14].

In order to identify if Case B had been exposed via aerosols in the wind blowing from the direction of the waste water treatment plant, meteorological data for the period of his working hours were obtained from a local weather station situated 1200 meters from the waste water treatment plant.

### Case A

Case A (male, 51 years, previously healthy, smoker) fell ill with pneumonia on August 9, 2006. Five days earlier, he had been accidentally exposed to aerosols of waste water, when water splashed during the installation of a new pump to the post-clarification basin. This basin separates water and sludge after the active sludge basin. The employees had been instructed to use respirators while working in the vicinity of the water treatment plant. Compliance with these instructions, however, was not good, and during the aerosol exposure, Case A had not been wearing a respirator.

Case A was diagnosed with legionellosis by urinary antigen immunoassay, this giving a positive response on the 7^th ^day. The first serological tests displayed an antibody response to *L. pneumophila *(the 6^th ^day, IgM++ and IgA+; the 23^rd ^day, IgM+++, IgA+++, IgG+++) and *L. micdadei *(the 6^th ^day, IgM+; the 23^rd ^day IgM++, IgG+, in-house EIA). The last test was conducted ten weeks after the onset of illness with the in-house IFA, giving values of 1:256 for *L. pneumophila *serogroup 1 and 1:1024 for *L. dumoffii*, while other *L. pneumophila *serogroups and *Legionella *species had a titer 1:128 at their highest. Despite repeated cultures no clinical isolate was ever obtained. Medical treatment was started two days after the onset of symptoms and was initially based on cefuroxime, roxithromycin, meropenem, but subsequently on piperacillin, and moxifloxacin was administered after the diagnosis of LD had been established. The patient spent four days in intensive care, and he was discharged after two weeks in hospital. This radiography-confirmed pneumonia case was classified as a confirmed case of LD according to EWGLI case definitions.

### Case B

Case B (male, 61 years, had stopped smoking five years previously) was diagnosed with pneumonia on September 24, 2006. He had been working about 200 m eastward from the active sludge basin and the cooling towers of the waste water treatment plant on days 4, 5, 6, 11, 12 and 13 before the onset of the respiratory symptoms. In the intervening days, he was not at work. This mill also instructed employees working in the vicinity of the water treatment plant to use respirators. However, Case B was not working at the waste water treatment plant and did not wear a respirator.

The diagnosis of LD for this radiography-confirmed case of pneumonia was based on serological tests. Nine days after the diagnosis, the patient tested seropositive for *L. pneumophila *serogroups 1-4 (IgM++, IgA+++, IgG+) and *L. micdadei *(IgG+, EIA). A subsequent serum sample analysed with IFA in another laboratory revealed a high titre for *L. pneumophila *serogroup 2 (1:4096, the 26^th ^day) and it was still high even though declining (1:2048) 12 weeks after the onset of the illness. For *L. micdadei*, the titers were 1:256 and 1:256, and for other *L. pneumophila *serogroups and *Legionella *species the titer values were only up to 1:64 and 1:128 (the 26^th ^day and 12 week samples). The difference between the causative serogroup and the other serogroups and species was at least two titer steps, showing the immune response specific for *L. pneumophila *serogroup 2.

His urinary antigen test was negative when tested on the 36^th ^day. No culturing for legionellae from clinical samples was performed. He was treated with roxithromycin and he recovered at home. This pneumonia was classified as a presumptive LD case.

Neither of the two cases had travelled abroad during the incubation period.

### Results of environmental study

In plant A, in February 2006, 2.0 × 10^7 ^cfu/l of *L. pneumophila *serogroup 13 was isolated from the active sludge basin, and 1.0 × 10^4 ^cfu/l of *L. pneumophila *serogroup 3 from water circulating around the plant (Table 1). After Case A had been diagnosed, the plant was sampled again, and high concentrations of *L. pneumophila *serogroup 1 were isolated from water in the active sludge basin, and from water and sludge in the post-clarification basin. Three strains of *L. pneumophila *serogroup 1 isolated from the post-clarification basin water and sludge were subtyped and the strains belonged to the monoclonal subgroup Bellingham.

**Table 1:** Environmental findings from the workplace of Case A, plant A

Date (before or after infection)	Water systems sampled (sample type)	Temperature (°C)	*Legionella *concentration (cfu/l)	Type of *Legionella*
**28^th ^Feb 2006** **(before)**	Active sludge basin at the waste water treatment plant (water)	33.0	2.0 × 10^7^	*L. pneumophila *serogroup 13

**28^th ^Feb 2006** **(before)**	Circulating waste water at the waste water treatment plant (water)	35.0	1.0 × 10^4^	*L. pneumophila *serogroup 3

**29^th ^Aug 2006** **(after)**	Active sludge basin at the waste water treatment plant (water)	36.0	1.0 × 10^6^	*L. pneumophila *serogroup 1

**29^th ^Aug 2006** **(after)**	Post-clarification basin at the waste water treatment plant (water)	35.0	2.3 × 10^4^	*L. pneumophila *serogroup 1, subgroup Bellingham

**29^th ^Aug 2006** **(after)**	Post-clarification basin at the waste water treatment plant (sludge)	36.0	2.0 × 10^6 ^and 1.0 × 10^6^	*L. pneumophila *serogroup 1, subgroup Bellingham and *L. pneumophila *serogroup 13

The domestic water systems of Case A were studied on August 29, 2006. Four samples were taken from the shower (mixture of hot and cold water, 35.5°C), kitchen tap (hot water, 55.0°C), toilet tap (mixture of hot and cold water, 35.5°C) and hose used outside the building (cold water, 10.2°C). All these domestic water samples were *Legionella*-negative.

In plant B, the active sludge basin was first sampled in August 2005, and 3.0 × 10^7 ^cfu/l of *L. pneumophila *serogroup 5 was detected (Table 2). After Case B had been diagnosed, new samples from the active sludge basin contained high concentrations of *Legionella rubrilucens *(8.0 × 10^9 ^cfu/l) and repeatedly *L. pneumophila *serogroup 5 (4.3 × 10^7 ^cfu/l). Legionellae were also isolated from a well where the rejected waste water was directed. In addition, a cooling tower at the water treatment plant, used for cooling of waste water, was sampled and yielded low concentration of *L. pneumophila *serogroup 2 (1.7 × 10^3 ^cfu/l). A few samples were also taken in November inside the mill, from paper machines and the shower used by Case B, but they did not contain culturable legionellae (Table 2).

**Table 2:** Environmental findings from the workplace of Case B, plant B and mill B

Date (before or after infection)	Water systems sampled (sample type)	Temperature (°C)	*Legionella *concentration (cfu/l)	**Type of *****Legionella***
**16^th ^Aug 2005** **(before)**	Active sludge basin at the waste water treatment plant B (water)	37.7	3.0 × 10^7^	*L. pneumophila *serogroup 5

**15^th ^Nov 2006** **(after)**	Active sludge basin at the waste water treatment plant B (water)	35.2	4.3 × 10^7 ^and 8.0 × 10^9^	*L. pneumophila *serogroup 5 and *L. rubrilucens*

**15^th ^Nov 2006** **(after)**	Cooling tower at the waste water treatment plant B (water)	37.3	1.7 × 10^3^	*L. pneumophila *serogroup 2

**15^th ^Nov 2006** **(after)**	Well of rejected waste water at plant B (water)	31.0	3.2 × 10^7 ^and 2.0 × 10^8^	*L. pneumophila *serogroup 5 and *L. rubrilucens*

**15^th ^Nov 2006** **(after)**	Process water, paper machine X, mill B (water)	50.0	Not detected	

**15^th ^Nov 2006** **(after)**	Process water, paper machines X and Y, mill B (water)	35.1	Not detected	

**15^th ^Nov 2006** **(after)**	Shower water system in mill B, used by Case B (water)	35.0	Not detected	

The domestic water systems of Case B were studied on November 23, 2006. The samples taken from the shower (mixture of hot and cold water, 37.7°C), kitchen tap (hot water, 53.4°C) and toilet tap (cold water, 11.3°C) were *Legionella*-negative.

**Figure 1:**
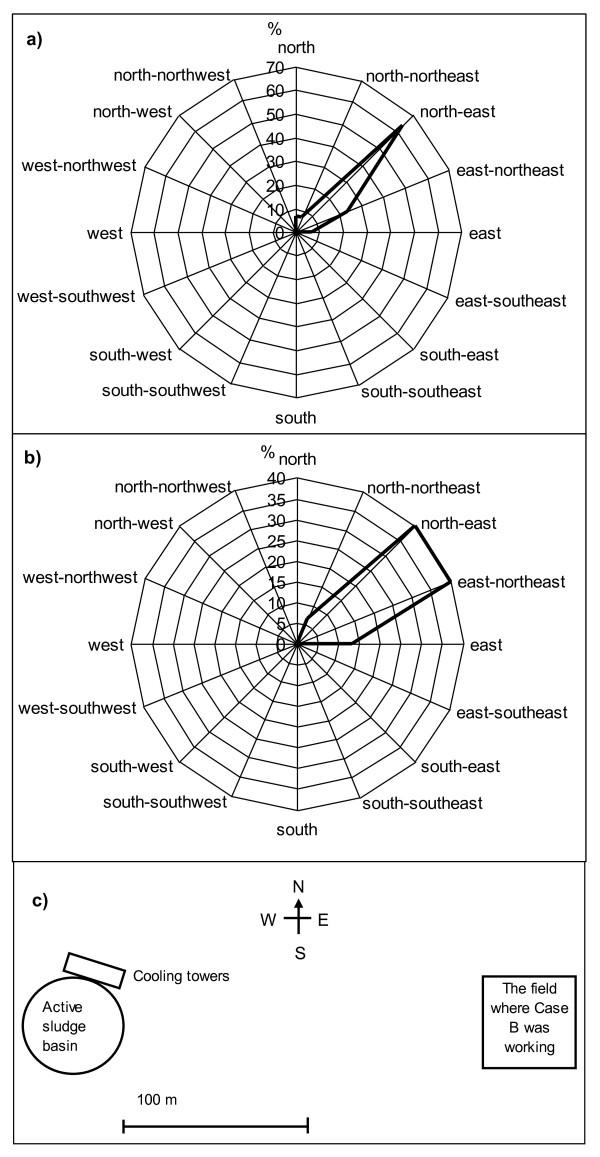
**Inverse wind roses (a, b) showing the direction where the wind had blown to, and location of the field where the Case B had been working, in relation to active sludge basin and cooling towers of the plant B (c)**. The wind data from the last working periods are drawn in separate inverse wind roses, a) is the wind data on days 4, 5 and 6 and b) on days 11, 12 and 13 days before the onset of symptoms in Case B.

Meteorological data reported that the wind had blown from south-west, west-southwest, south, south-south-west and west during the period of the latest working days and hours of Case B before the onset of symptoms. The wind speed was only from 1.1 to 4.2. m/s. This data from the local weather station, which was situated close to the waste water treatment plant B, was used to draw the inverse wind roses in Figure 1. Case B had been working in a field situated east from the waste water treatment plant B, and was exposed to the wind blowing exactly from west to east for at least four hours, 13 days before the onset of his symptoms.

## Discussion and Conclusion

This is the first report of LD cases associated with industrial waste water systems in Finland. In Case A, the positive urinary antigen test, and the antibody respose indicated that the infection was caused by *L. pneumophila *serogroup 1. The exposure to aerosols generated from waste water of the post-clarification basin was apparent and because *L. pneumophila *serogroup 1 was detected only in waste water samples, the most likely source of this *Legionella *infection was the post-clarification basin.

The antibody response of Case B suggested that LD was caused by *L. pneumophila *serogroup 2. This serogroup was isolated only from the cooling tower at the waste water treatment plant. In September 2006, the cooling towers were used occasionally, which was usual at that time of the year, meaning that a direct route via the cooling towers was possible. Water from the cooling towers flowed into the active sludge basin. Thus *L. pneumophila *serogroup 2 was very likely to be present in the active sludge basin, at least in low concentrations. However, it was not possible to detect by culture among the abundant growth of other *Legionella *strains and other microbes.

In 2007 and 2008, a total of 11897 LD cases were detected in Europe [2]. Most of these cases were diagnosed with UA test (81%) and only a few of the cases with the isolation of legionellae (8.8%). Thus it is very common that *Legionella *infections are diagnosed without *Legionella *isolates, which are needed for confirming the source. We were also unable to obtain clinical isolates in this study but were able to exclude some of the sources and focus on the most likely sources of transmission.

As Case B was working in a field about 200 m east of the active sludge basin and the cooling towers, meteorological data indicated that he was likely to have been exposed to aerosols originating from the waste water treatment plant. The incubation period of LD is generally between two and ten days. However, in the Dutch Flower Show outbreak in 1999, 16% of cases occurred after 10 days, up to 19 days [15]. In addition, in the 1976 Philadelphia outbreak incubation periods as long as 26 days were reported [16]. In Case A, the incubation time was five days. In Case B, the exact incubation period remained uncertain, as he had been working during the previous two weeks (days 4, 5, 6, 11, 12, 13) before the onset of symptoms under similar conditions. However, particularly on day 13, before the onset of symptoms, the wind was blowing for four hours from the waste water treatment plant exactly in the direction in which he was working. It is therefore possible that the incubation period exceeded 10 days, being up to 13 days in his case. After the domestic water systems and other water systems in the mill were excluded as possible sources, the waste water treatment plant remained the most likely source of the *Legionella *infection in Case B.

It is notable that even though very high concentrations of *L. rubrilucens *and *L. pneumophila *serogroup 5 were found in the waste water system of plant B, the antibody response indicated that he was suffering a *L. pneumophila *serogroup 2 infection; this serogroup was detected in much smaller concentrations at the plant. According to EWGLI data of LD cases from 1995 to 2006, 0.8% (33/4390) of the clinical *Legionella *isolates were *L. pneumophila *serogroup 2 [personal communication, Carol Joseph, 2009, EWGLI data, Health Protection Agency, London, England]. Equally rare were isolations of serogroup 5 of *L. pneumophila *(1.0%), in comparison to the isolations of serogroup 1 (73.8%), serogroup 3 (3.8%) and serogroup 6 (2.2%). In addition, *L. rubrilucens *has been known to be a cause of LD only once [17], showing that the species probably is much less virulent than all serogroups of *L. pneumophila*. The antibody response to *L. pneumophila *serogroup 2 does not rule out the possibility that the infection could have been caused by other legionellae, especially by those *L. pneumophila *serogroups prevailing in the waste water plant. Previously, *L. pneumophila *serogroup 5 has been associated with an outbreak of nosocomial legionellosis in Finland [6].

The dose of *Legionella *cells causing LD is not known precisely, nor how long one has to breath air with aerosols contaminated by legionellae before becoming infected. In the large outbreak in Pas-de-Calais, spending over 100 minutes outdoors daily increased the risk of LD significantly [18]. Prior to the onset of symptoms, Case B worked for at least 240 minutes (on day 13) downwind of the waste water treatment plant.

The laboratory results and environmental evidence of these two cases indicated that the LD infections were acquired at work, at or very close to the waste water treatment plants. No other previous or simultaneous *Legionella *infections have been known to occur among employees working at plants A or B, even though equally high *Legionella *concentrations most probably have existed in these waste water systems for years. The employees have worn respirators as protection against legionellae since 2005, and this may have prevented infections. Another explanation for the low number of cases could be underdiagnosis, since industrial waste water systems have been associated with abundant *Legionella *growth only since the Pas-de-Calais outbreak in 2003-2004 [18]. Therefore these waste water systems have not been investigated as possible sources of *Legionella *infections. Furthermore, the monoclonal subtype Bellingham of *L. pneumophila *serogroup 1 found in the plant A, does not belong to MAb 3/1 subgroup, which is the most virulent subtype of serogroup 1 [19], and *L. pneumophila *serogroups 2 and 5 rarely cause LD.

The two cases reported here have similarities to the previous Swedish case, where an employee of a paper and pulp mill most likely acquired infection while working 100 meters from a waste water treatment plant [7]. In that case, the clinical *Legionella *strain (*L. pneumphila *serogroup 1 subtype Benidorm, MAb 3/1 positive) and the environmental strain from the waste water basin were identical by molecular typing. In addition, an outbreak of five cases of Pontiac fever occurred after exposure to aerosols from sludge in a sewage treatment plant of the Danish food industry [20]. The strain isolated from sludge in concentrations of 1.5 × 10^7 ^cfu/g was *L. pneumophila *serogroup 1, subgroup OLDA/Oxford (MAb 3/1 negative). In this outbreak, the workers used respirators, but the filters were effective only against chemical substances.

Previously, a large community outbreak with 86 cases of LD was associated with cooling towers and waste water basins of a petrochemical plant occurred in France in 2003-2004 [18]. The strain causing the outbreak was *L. pneumophila *serogroup 1, strain Lens. The aerosols with legionellae, spread by a cooling tower, were infectious at a distance of at least six kilometers. The plant was later closed. In Norway, an air-scrubber was spreading *Legionella *aerosols over a distance of ten kilometers, resulting in 103 LD cases and ten deaths [21,22]. It is assumed that aerosols containing *Legionella *from the waste water aeration ponds originally contaminated the air scrubber. The scrubber was cleaned of legionellae and the aeration pond changed to an anaerobe pond. Thus, the sources of these larger outbreaks have been limited.

In contrast, Swedish and Finnish studies have indicated that heavy contamination of active sludge basins with legionellae is very common [7], (Unpublished data, Kusnetsov J, Torvinen E, Lehtola M and Miettinen IT). Further, air samplings in Norway and France in the vicinity of active sludge basins revealed that viable *Legionella *cells can be isolated up to 180-270 meters downwind [23,24]. It seems therefore likely that any waste water treatment plant with an active sludge basin under aeration can contain higher concentrations of *Legionella *bacteria and also produce aerosols with legionellae. Evidence of exposure to legionellae can be established if an increased frequency of elevated *Legionella *antibodies in serum samples can be detected [22].

In addition, some of these waste water treatment plants use cooling towers to lower the waste water temperature. It would be very useful to know if these waste water cooling towers are clean and maintained in accordance with the European and WHO *Legionella *guidelines [11,25]. In the European guidelines, a concentration of 1000 cfu/l of legionellae is recommended as the highest acceptable concentration which can be present in cooling water (technical guidelines).

Water treatment plants with active sludge basins should be considered as a possible source of community acquired *Legionella *infections, directly or indirectly via cooling towers. In addition, the employees should protect themselves by using respirators at or in the vicinity of water treatment plants. The Finnish Work safety act (738/2002) [26] has stated that employees should be protected against biological factors, including *Legionella *bacteria, at waste water treatment plants. Especially in industrial waste water treatment plants with high water temperatures, *Legionella *concentrations may be very high. In 2005, Finnish forest industry employees were instructed to use respirators while working in the vicinity of the water treatment plants. Developing ways to lower *Legionella *concentrations in these water systems would be the next step in diminishing the risk of *Legionella *infection.

These LD cases might have remained undiagnosed if our environmental study had not increased awareness about potential *Legionella *exposure in waste water treatment plants. These findings suggest that the clinicians should consider LD when treating patients with pneumonia from these industrial settings.

### Consent

Written informed consents were obtained from the Cases for publication of this report.

## Competing interests

The authors declare that they have no competing interests.

## Authors' contributions

JK arranged environmental samplings, interviewed the patients, wrote the first version of the manuscript, and had final responsibility for the decision to submit for publication. L-KN and TK were the physicians who cared for the cases, SAU and SM did typing of *Legionella *strains, and TP, TMNN and K-PM collected data. All the authors revised the article and approved the final version.

## Pre-publication history

The pre-publication history for this paper can be accessed here:

http://www.biomedcentral.com/1471-2334/10/343/prepub
